# xRatSLAM: An Extensible RatSLAM Computational Framework

**DOI:** 10.3390/s22218305

**Published:** 2022-10-29

**Authors:** Mauro Enrique de Souza Muñoz, Matheus Chaves Menezes, Edison Pignaton de Freitas, Sen Cheng, Paulo Rogério de Almeida Ribeiro, Areolino de Almeida Neto, Alexandre César Muniz de Oliveira

**Affiliations:** 1 LACMOR, Federal University of Maranhão, Av. dos Portugueses, 1966, São Luís 65080-805, MA, Brazil; 2 INF, Federal University of Rio Grande do Sul, Av. Bento Gonçalves, 9500, Porto Alegre 91501-970, RS, Brazil; 3 INI, Ruhr University Bochum, 44801 Bochum, Germany; 4 ECP, Federal University of Maranhão, Av. dos Portugueses, 1966, São Luís 65080-805, MA, Brazil; 5 DEINF, Federal University of Maranhão, Av. dos Portugueses, 1966, São Luís 65080-805, MA, Brazil

**Keywords:** robotics, simultaneous localization and mapping, RatSLAM, image SLAM

## Abstract

Simultaneous localization and mapping (SLAM) refers to techniques for autonomously constructing a map of an unknown environment while, at the same time, locating the robot in this map. RatSLAM, a prevalent method, is based on the navigation system found in rodent brains. It has served as a base algorithm for other bioinspired approaches, and its implementation has been extended to incorporate new features. This work proposes xRatSLAM: an extensible, parallel, open-source framework applicable for developing and testing new RatSLAM variations. Tests were carried out to evaluate and validate the proposed framework, allowing the comparison of xRatSLAM with OpenRatSLAM and assessing the impact of replacing framework components. The results provide evidence that the maps produced by xRatSLAM are similar to those produced by OpenRatSLAM when they are fed with the same input parameters, which is a positive result, and that implemented modules can be easily changed without impacting other parts of the framework.

## 1. Introduction

Simultaneous localization and mapping (SLAM) deals with the robotic problem of autonomously building the map of an initially unknown environment at the same time as it locates the robot on this map. The map defines the robot’s localization by a pose (position and orientation). The map can be understood as an abstract representation of a set of resources describing the environment, such as walls, obstacles, landmarks, and so on [1]. Therefore, a robot running SLAM incrementally builds a representation of the environment through pose estimations obtained from the data collected by its sensors [2].

Besides these more general SLAM approaches, some bioinspired algorithms have also been proposed for some specific autonomous robotic applications, such as exploration of hazardous areas [3,4], robotic area surveillance [5], and robotic search and rescue [6,7,8].

One such bioinspired SLAM algorithm is RatSLAM [9], which was inspired by the navigation system found in rodent brains, where the hippocampus and entorhinal cortex play an important role in spatial navigation [10,11,12]. RatSLAM works both in indoor and outdoor environments and requires only a monocular vision sensor [9,13,14,15,16]. Several RatSLAM-based algorithms have been proposed recently, demonstrating continued interest in bioinspired approaches for both robotics and neuroscience applications [17,18,19].

The RatSLAM algorithm has three main computational modules: LocalView, PoseCell and ExperienceMap. The LocalView module processes the robot’s camera inputs. The PoseCell module keeps track of the robot’s pose over time, combining odometry (path integration) and sensor information from the LocalView module. The ExperienceMap module collects experiences and embeds them into a topological graph representing the environment’s topology.

The work reported in [15] proposed a MatLab implementation of RatSLAM and demonstrated how a rat animat robot could learn a spatial layout, including the ability to close physical loops based only on visual input (image sequences) and robot odometry (dead reckoning). However, this implementation was not designed to be used by different programmable robot interfaces since it was too slow to be applied in real-time for large environments [16].

OpenRatSLAM, proposed in [16], is an open-source RatSLAM implementation based on the Robot Operating System (ROS) [20], widely used in robotics. OpenRatSLAM implements each module as an ROS node inheriting the ROS intrinsic node parallelisation and the ability to integrate more than one robot architecture. However, OpenRatSLAM cannot be used as a library since it cannot be easily integrated as a module by other computational applications.

Therefore, the C++ library libratslam was developed [21], which employed a couple of threads to run RatSLAM modules in parallel and could be easily integrated with other computational systems. A drawback of that library was that it was not easy to modify its modules’ code to implement another biological concept, as the classes that implemented the RatSLAM modules were not designed as classes expected to be specialised.

### 1.1. Main Objective

In this work, we propose xRatSLAM, an extensible, parallel, open-source framework (code available at [22]) intended to facilitate the development and testing of new RatSLAM algorithm variations, allowing researchers and developers to share RatSLAM modules code and compare their experiment results easily. The xRatSLAM framework was conceived so that each RatSLAM module could be recoded without needing to change any other module, i.e., applying good practices to achieve high cohesion and low coupling. Furthermore, by mixing the features of a framework and a library, xRatSLAM allows us to dynamically choose different module versions or biological behaviours.

Researchers have proposed libraries and open-source implementations to reduce unnecessary self-research (or knowledge recycling). A recent example is an augmented reality system design that can be used in mobile devices [23] based on the previously proposed OpenVSLAM [24]. The development of open-source libraries and frameworks is justified whenever there is a growing interest by the academic community in responding to scientific and technological demands, such as new propositions of methods for bioinspired mapping or even immersion in indoor augmented reality [23].

### 1.2. Contributions and Organisation

The main contribution of this work is an open-source, modular implementation of RatSLAM mainly featured by:

- Processing: suitable to produce maps from batch and real-time image streams;
- Flexibility: developers can focus on implementing all or some specific RatSLAM components, allowing them to use third part modules;
- Library: an efficient, easy-to-use, and integrated C++ library;
- Compatibility: compatible with well-settled RatSLAM implementations by using the same input and output;
- Debugging: easy access to specific RatSLAM internal records for logging, tracing, and monitoring tasks.

This work also contributes to the literature on RatSLAM both in the conceptual and practical fields. It is essential to mention that the technical specification of the proposed piece of software is also a relevant result as it allows other developers to design new frameworks and tools for bioinspired mapping and localization.

This paper is divided into sections as follows: Section 2 presents an overview of the main RatSLAM concepts and algorithms. In Section 3 and Section 4, the proposal is detailed. Section 5 highlights the main experiments designed to evaluate and validate the proposed framework. Finally, in Section 6, conclusions and new directions are summarised and discussed.

## 2. The RatSLAM Dynamics

RatSLAM is a SLAM system inspired by computational models of the neural processes underlying navigation in the hippocampus and the entorhinal cortex of rodents [9]. Over time, RatSLAM has been enhanced to work with general real-world examples of localization and mapping using a video camera as its main input sensor [13,25,26]. Since RatSLAM has been discussed extensively in the literature (e.g., [9,14,16]), we only briefly describe the system in the following.

**Figure 1:**
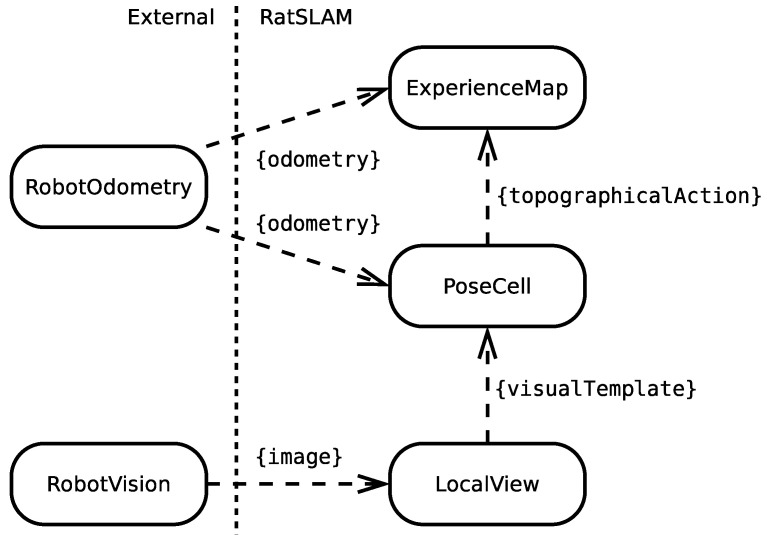
The main RatSLAM modules: LocalView, PoseCell, and ExperienceMap, including the external modules RobotOdometry and RobotVision. Dashed arrows indicate messages.

The RatSLAM architecture is composed of three main modules: PoseCell, LocalView, and ExperienceMap. The system uses two external modules to capture input information: the RobotVision module, which captures camera images of the environment, and the RobotOdometry module, which captures robot self-motion cues. Figure 1 shows RatSLAM’s internal and external modules and their interactions.

An overview of the three RatSLAM original modules’ structures and behaviours is presented in the next section. A more detailed algorithm description of the RatSLAM and its three modules can be found in [9].

### 2.1. PoseCell Module

The PoseCell is a continuous attractor network (CAN) configured in a three-dimensional matrix with fixed hard-coded weights between its cells. The weights are set to excite nearby cells and inhibit distant ones. This structure aims to keep track of clusters of excitatory cells called activity packets. The network’s total activity level is kept constant. Hence, as distant cells inhibit each other, distant activity packets compete with each other. By contrast, the excitation between nearby cells fuses activity packets near each other. The network uses periodic boundary conditions, i.e., the cells on the network’s boundary are neighbours.

The PoseCell elements are not meant to represent each possible robot pose. Therefore, the possible robot pose representation is limited by PoseCell dimension sizes. Instead, the PoseCell network serves to resolve conflicts between competing sensor readings. The activity packet remains stable in the absence of external excitation. Two external inputs can modify its dynamics: odometry readings and sensory data. The odometry indicates how the robot’s pose has changed at each time step. If the odometry can be trusted, this pose variation is used to find the PoseCell element capable of representing the next robot pose. Then, this element is injected with some excitation energy. The influence of the sensory data is discussed in the following section.

### 2.2. LocalView

Sensory data influence PoseCell elements through the LocalView module. The LocalView structure stores features of sensory data perceived at each robot pose and checks if the current sensor data are new or have been perceived before. In the original RatSLAM implementation, this structure stores image features in a list. Each time a new image is captured, its features are extracted and compared with all stored images using a match threshold parameter.

If the current sensory input is new, it is stored and associated with the PoseCell element representing the centre of the current activity packet. If the current input matches a stored image, its associated PoseCell element is excited. When repeated, the excitation of PoseCell elements by known sensory inputs leads to a growth of an activity packet in the PoseCell network, which competes with the existing activity packet maintained by robot odometry. The coherence of sensory data determines the winner of this competition.

### 2.3. ExperienceMap

RatSLAM was initially designed for use in a 2D space based on visual sensors in a terrestrial robot. Therefore, the topological map of the environment is stored as a list of nodes, called *experiences*, which store the robot poses and the elements of LocalView and PoseCell that were activated when they were created. The graph links store times and distances between the experiences.

Since the experience references the activated PoseCell and LocalView elements, it is possible to detect whether the robot is in a place it has occupied before. When this occurs, a loop is detected, and the path correction process takes place to reorganise all ExperienceMap node poses.

## 3. The Proposed Extensible RatSLAM Framework

The proposed xRatSLAM framework is designed to address computational issues in previous works and foreseen future needs, yielding the following guidelines:

1. xRatSLAM is implemented as a library, so it can be easily incorporated into different robotic applications;
2. Due to its modularity, it must be easy for developers to change the code implementation of any RatSLAM module without the need to recode any other modules or the external relations, as data input or output related ones;
3. The sensor generalisation provides a facility for integration with other sensor inputs beyond images;
4. The module implementations should run in multithreading mode, enabling parallelisation;
5. The framework input parameters are compatible with the configuration parameters used by the original RatSLAM implementation.

### 3.1. xRatSLAM as a Library Design

As xRatSLAM can be used as a C++ library, it can be ported to any C++/11 standard robotic compliant system. This approach was motivated by the possibility of subdividing complex problems into different components implemented in independent libraries: autonomous navigation, obstacle avoidance, learning the environment, mapping, etc. In this scenario, it is easier for the designer to build the robotic application using libraries dedicated to each specific robotic task.

### 3.2. xRatSLAM as a Framework

Framework modelling has a more direct impact on developing new features and behaviour. The advantage of the xRatSLAM framework stems from the definition of the system architecture, which is often the most challenging part of the software design, generally neglected by other reuse techniques. The modelling by framework simplifies the synchronisation of the execution of individual components and the communication among them. It also provides highly optimised and parallelised methods to transfer data between model grids [27].

### 3.3. xRatSLAM Modules

**Figure 2:**
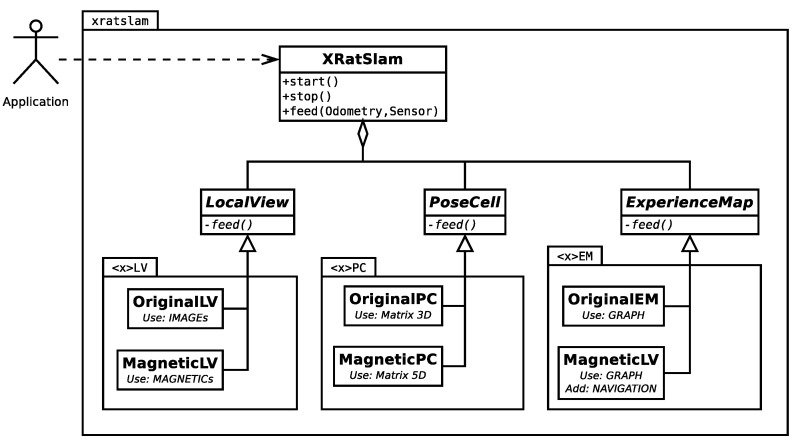
xRatSLAM main class diagram. Modules are designed as abstract classes, which different possible module implementations can easily overload. The diagram shows two implementation examples for the LocalView module: the original one uses visual sensors, and another uses magnetic sensors. Used symbols follow standard UML.

The xRatSLAM library interface was implemented through a single class named xratslam::XRatSLAM. The library class diagram is shown in Figure 2. The interface class allows the user to start and stop internal module threads, feed input data, read results, and perform other practical computing operations. The xRatSLAM framework uses one abstract base class for each RatSLAM module and the original proposed modules are implemented as classes extending its respective base class OriginalLV, OriginalPC, and OriginalEM (Figure 2).

This way, the original RatSLAM modules are seen as examples of how the modules can be implemented, allowing developers to create their module implementations. For instance, LocalView uses generic sensor data instead of the image data used by the original algorithm (OriginalLV). This modification allows, for example, the implementation of a MagneticLV to deal with magnetic or another kind of sensor [28].

An important benefit of the proposed architecture is that it lets users choose dynamically which combination of module implementations they would like to use. This is done through the xRatSLAM methods: setLocalView(), setPoseCell(), and setExperienceMap().

### 3.4. Module Parallelisation

**Figure 3:**
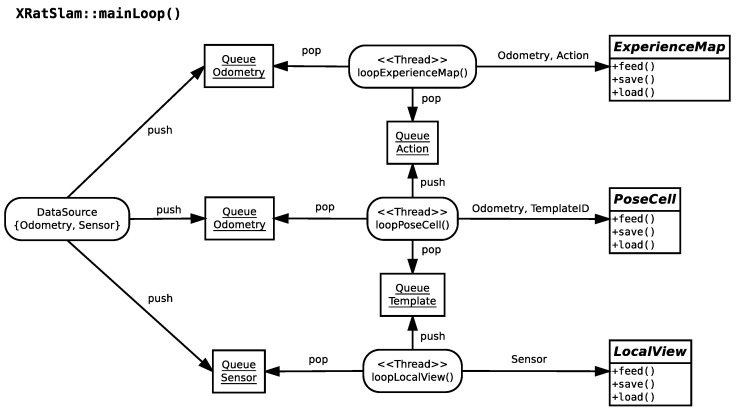
The xRatSLAM main execution loop. The main loop and internal threads communicate using data queues. Incoming odometry and sensor data are stored in its respective queue (Odometry is replicated). Each module (LocalView, PoseCell, and ExperienceMap) has its own thread responsible for reading incoming queue data and communicating with its respective module abstract class to process the data and then store the result in a queue for further processing. Arrows indicate dependencies between objects.

The OpenRatSLAM implementation uses parallelisation that is inherent in ROS nodes [16]. In ROS, each node runs as a different system process, synchronised through the ROS topic-publishing mechanism. By contrast, xRatSLAM parallelisation is implemented through different threads of the same process. Each thread runs on an internal loop method responsible for reading data from one or more synchronised queues, calling the associated module entry point in its abstract class (Figure 3).

The synchronisation between threads is achieved through data queues. For example, consider the case when loopExperienceMap() tries to read data from QueueOdometry. If the queue is empty, the thread is blocked until some Odometry data are inserted in the queue.

Once data are available, the thread tries to read an Action object from QueueAction, and, again, it is blocked until there are data to be read. When both Odometry and Action are read, the thread calls ExperienceMap::onFeed() to let the ExperienceMap module perform its computations. The loop restarts, and the thread tries to read the next QueueOdometry data.

All threads are also blocked when trying to insert an element into a full queue. The queue’s push-and-pop blocking mechanisms ensure synchronisation among the internal loop threads. The maximum size of all queues is hard-coded and is set to 10 elements as this size was empirically found to be sufficient for obtaining satisfactory parallelisation results.

### 3.5. Customised Modules

**Figure 4:**
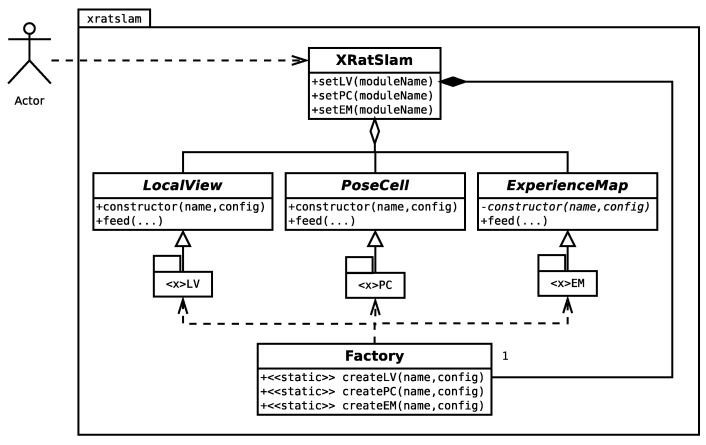
xRatSLAM class diagram highlighting factory project aspects. The Factory design pattern allows for dynamically instantiating each specific module implementation based on its respective name by knowing internal module packages independently of package implementations. Note that the system uses just one Factory element. Used symbols follow standard UML.

Customised RatSLAM modules can be internally instantiated using the Factory design pattern (Figure 4).

Currently, there is no *plug-and-play* mechanism to insert a new module into the framework. Thus, the following steps are required:

1. Create a class with the new module code extending the corresponding module base class (LocalView, PoseCell, or ExperienceMap). The derived class must implement at least the base class pure virtual methods.
2. Change the Factory class code, so it knows how to instantiate the newly created module by name.
3. As xRatSLAM uses cmake to build the code, all *.cc used by the new module should be added to "CMakeLists.txt” file.
4. Finally, the client program should select the customised module, by its name (as a xRatSLAM object), before calling XRatSLAM::start() as explained in Section 4.

All modules are accessed by calling their onFeed() method, which receives different parameters for each module and has the following functions:

- LocalView: receives a Sensor object (ex: Image) and generates a templateId number identifying a sensory scene corresponding to the given sensor object. If the object was already perceived, the templateId should be set to the id of the previously sensed object. If not, a new template should be created with a new templateId.
- PoseCell: receives an Odometry and a Template object representing what is currently sensed and generating an Action object. The module should decide where the agent stores the log of odometry data and experiences. The Action indicates what should be done by the ExperienceMap: (a) do nothing, (b) store a new pose, or (c) update a pose already known.
- ExperienceMap: receives an Odometry and an Action object. It uses Odometry to update a guess about the physical location and Action to create new location landmarks or to link existing ones.

### 3.6. Using Different Types of Sensors

In RatSLAM, loop closures are triggered by the LocalView module whenever it detects that the current state has been sensed. In the original implementation, an image template is created to represent a set of similar images. Each new template is compared to the stored ones. The current template is discarded, and loop closure is triggered if already sensed. Otherwise, the current template represents a new state and should be stored in memory for later comparisons. Hence, a sensed state creation action is generated. Since RatSLAM is not intrinsically restricted to using only images and can use other data, it is worth mentioning that LocalView is the only module directly affected by the type of sensor used. The proposed framework generalises sensor types using the Sensor class as the base class for all implemented sensor types.

### 3.7. Development Aspects

xRatSLAM is designed to be used in production systems, algorithm development, and test environments. When testing a new module implementation or comparing performances of different module implementations, it is desirable to log or even visualise some internal states and measure each module’s execution time performance. xRatSLAM provides three ways to assess how the algorithm modules perform.

- Execution time. A TimeLogger object can be set for each module. This object can be used to access the time measures in the corresponding module’s past executions. This metric measures only the execution of the module onFeed() method, excluding the time spent waiting for module input data to become available.
- Internal state. Access to internal data structures is desirable to provide users with graphical feedback on what is happening while the algorithm is running. From the framework point of view, it represents a challenge because the framework is unaware of the modules’ internal data structures. The current framework implementation, therefore, imposes a semantic data structure for each module. This way, LocalView implementations should return a set of Template objects. PoseCell implementations shall return a 3D activation matrix, and ExperienceMap implementations shall return a topological graph. Note that data access interfaces must be changed if a new module implementation does not fit in.
- State storage. A common scenario in the algorithm development process is testing the code implementation on captured data, sometimes using a benchmark data set. This process can be inefficient when long-running experiments and errors only occur later. The xRatSLAM class has an interface to save() and load() the modules’ internal states. Each module extension should implement such methods. If so, the xRatSLAM algorithm can be restored by saving the algorithm state and restarting its execution from that point.

### 3.8. Peripheral Computational Tools

All RatSLAM implementations share some basic computational modules for general tasks unrelated to the algorithms. These peripheral modules play an essential role in the framework architecture. How these modules are designed determines how easily the framework can adapt to a new situation. There are two crucial peripheral modules used to transform how data are captured.

- Sensor reader. The framework is meant to be used by a robot capable of reading its sensory and odometry data in real time from its hardware, but it is also meant to be used by researchers whose experimental data are stored in files, e.g., on a desktop computer. Therefore, the peripheral DataReader class can read data directly from sensors or files that store previously recorded sensor data.
- Visual odometry. The RatSLAM algorithm was conceived to use robot odometry and sensory image data. However, the odometry information can be extracted from a sequence of images. This module can use those images to generate visual-based odometry, replacing the robot odometry information as proposed by [9].

## 4. xRatSLAM Usage

The xRatSLAM parameters are read from a configuration file following the same syntax suggested in [16]. The basic algorithm usage is shown in Listing 1. Note that the client program can select which combination of LocalView, PoseCell, and ExperienceMap modules are used. Default modules are also available following the implementation used in [16].

The way to obtain Sensor and Odometry input data is not a concern for xRatSLAM. However, to support developers, two classes are made available to read image sequences from video files (DataReaderVideo) and to read from multiple image files (DataReaderDir). These classes can also read Odometry data from an odometry file or generate odometry information from image sequences (visual odometry). Note that the application can read these input data directly from a robot.

The client application controls the program flow by calling the start() and stop() methods to start and stop all loop threads, respectively. At each iteration, the application reads Sensor and Odometry input data and feeds them by the XRatSLAM::feed() method. When the stop() method is called, all threads ensure that the data of all synchronised queues are properly processed before they terminate.

Finally, line 21 illustrates that the client can optionally read some internal data and use them, for example, to show graphical feedback to end users.

**Listing 1.** Simple pseudocode to illustrate xRatSLAM usage.
1xRatSLAM slam( configFile )2 3slam.setLocalView    ( moduleName )      // Optional.4slam.setPoseCell     ( moduleName )         // Optional.5slam.setExperienceMap( moduleName )  // Optional.6 7// Read input images from video file.8DataReaderVideo reader( configFile, videoInput );9 10// Use visual odometry.11reader.setOdometryVisual( configFile );12 13TimeLogger logger( logDir );        // Optional.14slam.setLogger( logger )                // Optional.15 16slam.start()      // Start all xRatSLAM~threads.17 18while ( reader.readNext( Sensor, Odometry ) )19{20  slam.feed( Sensor, Odometry )21  drawEM( slam.getDataEM() )         // Optional.22}23 24slam.stop()         // Stop all xRatSLAM threads.


## 5. Experiments

This section presents three experiments that were performed to evaluate and validate the proposed framework. The first one is meant to compare the maps and running times obtained by xRatSLAM to those of OpenRatSLAM, both running standard modules. The second experiment evaluates the flexibility of xRatSLAM, since any module implementation can be changed without impacting any other part of the framework. Finally, the third experiment compares the data collected by visual and wheel odometry, i.e., a typical RatSLAM experiment that usually shows the behaviour of the peripheral modules.

We ran all experiments on an Intel(R) Core(TM) i7-2630QM CPU @ 2.00 GHz, one physical processor, four cores, eight threads with 4G of RAM running the Debian GNU/Linux 11 (bullseye) operating system.

### 5.1. Experiment I

Performance requirements concern the framework’s accomplishment of certain functions under specific conditions. They can be evaluated by measuring response and running times and storage requirements. In this section, the xRatSLAM framework is compared to OpenRatSLAM in terms of running time for mapping under the same circumstances, i.e., the same data input.

For fairness, the code used by OpenRatSLAM for all three RatSLAM modules (LocalView, PoseCell, and ExperienceMap) was inserted as modules in the xRatSLAM framework to run both programs with the same inputs and module implementations.

The kits *iRat* and *Robodeck* [16,21] were used to compare both implementations. The iRat (Intelligent Rat Animat Technology) robot (www.davidmichaelball.com/portfolio-items/irat-intelligent-rat-animat-technology/ accessed on 20 October 2022) is designed for studies in navigation, embodied cognition, and neuroscience research, equipped with a camera sensor, proximity, and odometry sensors in the wheels. The Robodeck (http://www.xbot.com.br/educacional/robodeck/ accessed on 20 October 2022) is a wheel-based mobile robotic educational platform composed of four independent wheels, each controlled by a steering servo motor. The data generated by Robodeck consist of a video stream collected by a low-resolution camera during an indoor tour in a research laboratory [21].

**Figure 5:**
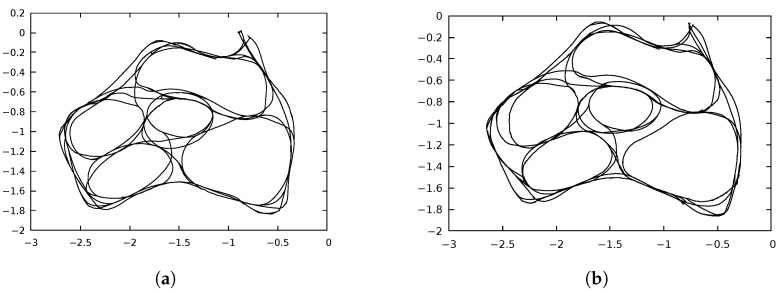
ExperienceMap generated in *iRat* experiment. ICP error obtained was 0.0117619. (**a**) *iRat* robot path found by OpenRatSLAM. Time: 960 s. (**b**) *iRat* robot path found by xRatSLAM. Time: 245 s.

**Figure 6:**
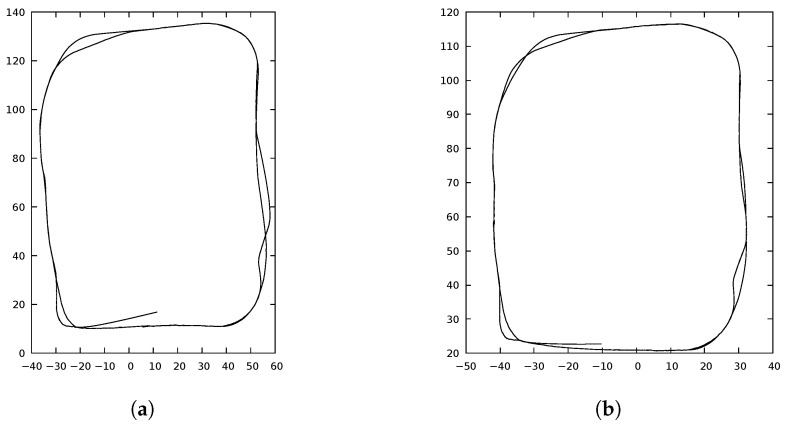
ExperienceMap generated in Robodeck experiment. ICP error obtained was 0.488817. (**a**) Robodeck robot path found by OpenRatSLAM. Time: 204 s. (**b**) Robodeck robot path found by xRatSLAM. Time: 38 s.

The experience maps generated by the two implementations were very similar, but they were not identical. The experience maps were compared using libicp software [29], which implements the iterative closest point (ICP) algorithm. Figure 5a,b show the resulting ExperienceMaps generated by the OpenRatSLAM and xRatSLAM implementations, respectively, for the iRat experiment. Similarly, Figure 6 shows the results for the Robodeck experiment. The error obtained by ICP were 0.0117619 (iRat) and 0.488817 (Robodeck). Interestingly, the more complicated complex mapping had the lowest ICP error.

The divergence could be explained by the fact that OpenRatSLAM is based on the Robot Operating System (ROS) (https://www.ros.org/ accessed on 20 October 2022), which does not guarantee reading all input data in the same sequence as xRatSLAM does [20]. Figure 6a was directly generated by the OpenRatSLAM plot procedure, which does not preserve the *x*:*y* ratio.

The run time required by OpenRatSLAM was about 960 s, while xRatSLAM took only about 245 s to obtain the ExperienceMap for the iRat data set. Considering the running times for generating the experience maps of the two video data (iRat and Robodeck), xRatSLAM tended to be around four to five times faster than OpenRatSLAM.

Regarding the time analysis, it is essential to mention that the input data were the same for both software executions and that both implementations were deterministic. Therefore, only one execution was sufficient for collecting and analysing the running times.

The results could ensure that, under the same circumstances, xRatSLAM reached similar experience maps faster. It is worth mentioning that whilst OpenRatSLAM ran with a parallel process, xRatSLAM ran with parallel threads. Moreover, the improvements in parallelisation combined with some small module recoding were responsible for the gains concerning running time.

**Figure 7:**
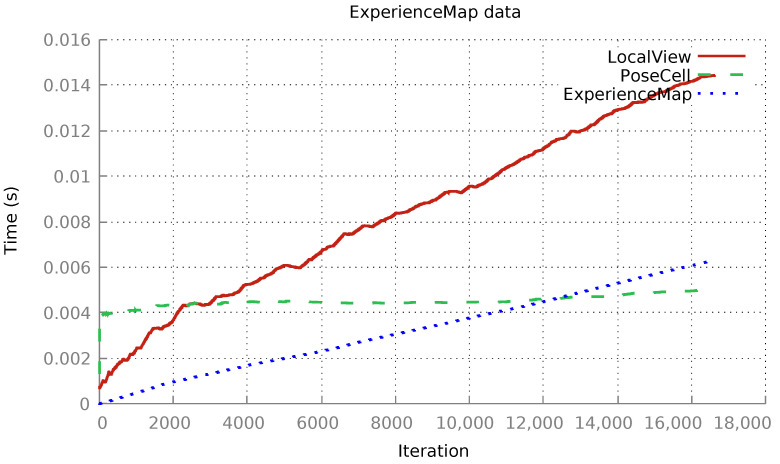
The iRat experiment analysed by xRatSLAM. Modules execution time over iterations: LocalView average: 14.43 ms. PoseCell average: 4.99 ms. ExperienceMap average: 6.31 ms. Total time: 245 s.

Furthermore, the run-time measures of each xRatSLAM module were further analysed using tracing features available in the framework. The separate performance evaluation identified which modules were more sensitive and required for eventual implementation improvements. Figure 7 shows the average running time for each module in each iteration step. While the PoseCell execution time per iteration was nearly constant, the LocalView and ExperienceMap running times grew linearly as the template list became longer.

Analysing the RatSLAM modules revealed that the LocalView module had poor performance. This module stores image templates of image views that were encountered in the environment. When a new image is perceived, its template is generated and compared to all stored visual templates, as discussed previously. The linear increase in the running time of LocalView (Figure 7) can be explained by the linear search algorithm used in this module, i.e., the search for a matched template is started at the first stored template and proceeds linearly through the list of stored templates.

### 5.2. Experiment II

As observed in Figure 7, the running time of this module had a linear asymptotic growth. A small change in the search algorithm’s implementation in LocalView might significantly impact performance. The new LocalView implementation started at the last template and moved backward through the template list. If the template was stored in the list, the running time became mainly independent of the list size because it was very likely that a known template had been encountered recently. If the template was new, the search had to proceed through the entire list, and the algorithm obviously depended on the list size, and the running time scaled linearly.

Beyond the new implementation, this experiment was used to assess the impact of a simple code change and log resources available in the xRatSLAM framework. This experiment also demonstrated how the performance of a specific module could be measured and how a new module implementation could be added to the framework, focusing only on one module code.

**Figure 8:**
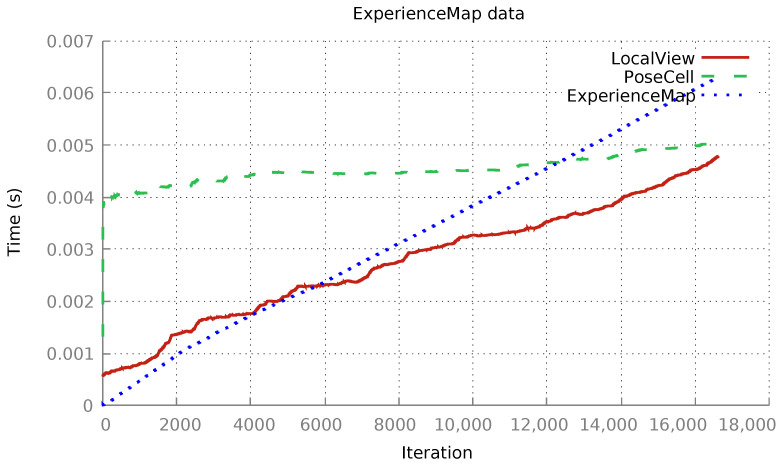
Average execution times of xRatSLAM using the RecodedLV module implementation. LocalView average: 4.79 ms. PoseCell average: 5.01 ms. ExperienceMap average: 6.30 ms. Total time: 121.0 s.

The xRatSLAM framework was executed using the new LocalView implementation, RecodedLV, for which the following average execution time was obtained (see Figure 8).

As expected, the average run time of the LocalView module still grew linearly, but it increased much more slowly for *RecodedLV*—notably the execution time of the new *RecodedLV* module was 4.79 ms as compared to 14.43 ms for *OriginalLV* module in Experiment I. The average execution times of both PoseCell and ExperienceMap were similar to the previous implementation (Figure 7). A single, small modification in the LocalView implementation cut the total execution time in half from 245.0 s (Figure 7) to 121.0 s (Figure 8). Hence, it is possible to interchange execution between any module implementations (see Listing 1) without needing to inspect or recode any other part of the system.

### 5.3. Experiment III

In this third experiment, the results obtained using wheel odometry were compared to those obtained with visual odometry based on changes between consecutive video frames. xRatSLAM used the same visual odometry code available in [16].

**Figure 9:**
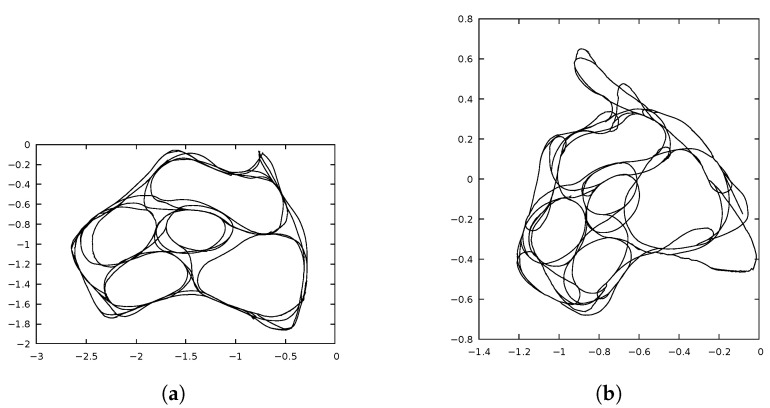
ExperienceMap comparison between different odometry sources. (**a**) ExperienceMap using odometry information read from *iRat* robot. (**b**) ExperienceMap using odometry information generated by visual odometry with parameters empirically tuned.

Figure 9a shows the resulting experience map obtained when the iRat robot generated the odometry, whilst Figure 9b shows the experience map obtained using the visual odometry, calculated by xRatSLAM. As expected, the visual odometry was not as good at generating closed curves as the robot odometry.

## 6. Conclusions and Future Work

This work proposed an extensible, parallel, open-source framework implementation of RatSLAM. This framework aimed to facilitate the collaborative development and testing of RatSLAM-based algorithms, allowing researchers and developers to easily share code for RatSLAM modules and compare their performance in experiments.

The xRatSLAM framework was designed to make it possible to recode or replace a module with no impact on other modules and apply good practices to achieve high cohesion and low coupling. Furthermore, by mixing the features of a framework and a library, xRatSLAM allowed us to dynamically choose different module versions or even biological behaviours. The modelling following the framework simplified the synchronisation of the execution of individual components and the communication among them. It also provided highly optimised and parallel data transfer between modules.

Three experiments were conducted to assess and validate the proposed framework. The results showed that:

(i) xRatSLAM could produce similar maps compared with OpenRatSLAM when fed with the same input data, running faster than OpenRatSLAM;
(ii) A module implementation could be easily changed without impacting any other part of the framework;
(iii) The framework was featured by logging capabilities that allowed a detailed analysis of the results;
(iv) In the absence of a robot mechanical generated odometry, visual odometry could generate good approximate maps for the case studied.

In the accuracy and performance contexts (item (i)), xRatSLAM has already been used by researchers in experiments that require shorter execution times to perform time-consuming tasks, such as parameter tuning in long-term mapping [30,31]. However, opportunities for enhancements are highlighted below to show how the framework can be improved:

(i) Other RatSLAM module implementations;
(ii) A built-in assessment module for mapping accuracy evaluation;
(iii) Dynamic libraries (plug-ins) in the module inclusion mechanism;
(iv) Support for 3D SLAM, as required for unmanned aerial vehicles (UAV: drones) or uncrewed underwater vehicles (UUV);
(v) A ROS wrapper for xRatSLAM;
(vi) An interface for other programming languages such as Python;
(vii) A module repository for sharing implementations between different users;
(viii) Usability improvements to suit neuroscience theorists and practitioners.

The fundamental point is that xRatSLAM is not a substitute for the OpenRatSLAM framework. The xRatSLAM is an alternative for robot systems using the RatSLAM algorithm as a library without ROS. Compared with OpenRatSLAM, the main drawback is that the xRatSLAM uses a library-like interface, so only a C++ code can access it. Moreover, xRatSLAM does not provide a mechanism to use stored input data respecting the real time they were saved at. As OpenRatSLAM uses ROS, this mechanism is available.

However, OpenRatSLAM uses the ROS node interface. Thus, not just the program accessing the RatSLAM needs to be written in any ROS-compliant language, but also its modules. Robot systems already using the ROS framework can easily adopt the OpenRatSLAM solution, while the use of xRatSLAM requires extra effort to create a ROS node to communicate with it.

## Data Availability

xRatSLAM code is available to download under a GPLv3 License at https://doi.org/10.17632/rrjv728cmj.1.
